# TBX3 Knockdown Decreases Reprogramming Efficiency of Human Cells

**DOI:** 10.1155/2016/6759343

**Published:** 2015-11-30

**Authors:** Moritz Klingenstein, Stefanie Raab, Kevin Achberger, Alexander Kleger, Stefan Liebau, Leonhard Linta

**Affiliations:** ^1^Institute of Neuroanatomy, Eberhard Karls University Tübingen, 72074 Tübingen, Germany; ^2^Department of Internal Medicine I, Ulm University, 89081 Ulm, Germany

## Abstract

TBX3 is a member of the T-box transcription factor family and is involved in the core pluripotency network. Despite this role in the pluripotency network, its contribution to the reprogramming process during the generation of human induced pluripotent stem cells remains elusive. In this respect, we performed reprogramming experiments applying *TBX3* knockdown in human fibroblasts and keratinocytes. Knockdown of *TBX3* in both somatic cell types decreased the reprogramming efficiencies in comparison to control cells but with unchanged reprogramming kinetics. The resulting iPSCs were indistinguishable from control cells and displayed a normal *in vitro* differentiation capacity by generating cells of all three germ layers comparable to the controls.

## 1. Introduction

Pluripotent embryonic stem cells (ESCs) are isolated from the inner cell mass of early embryos. Pluripotency is defined by a high and indefinite proliferative potential and the capacity to differentiate into cells of the three germ layers, subsequently able to generate all cell types and tissues of an organism. The possibility of culturing and investigating ESCs has led to a good understanding of the pluripotency network, but also of various signaling pathways involved in cell differentiation events [1, 2]. The pluripotency network is comprised of numerous transcription factors, interconnected with each other by regulatory feedback loops. Basically, it keeps the expression of stem cell genes active and represses genes involved in differentiation [3, 4]. These transcription factors have such a dominant role in cell function that the overexpression of a few factors is able to force a somatic cell back into a pluripotent state. In this way, somatic cells are reprogrammed into induced pluripotent stem cells (iPSCs) by overexpression of* OCT4*,* SOX2*,* KLF4,* or other related factors [5, 6]. The discovery of this reprogramming technique has not just widened the understanding of the pluripotency network but is also an invaluable tool to generate donor and patient specific stem cells for disease modeling and for future therapeutic strategies [7–12].

The T-box transcription factor family is involved in a variety of processes including differentiation and pluripotency. In the pluripotency circuitry, TBX3 has been shown to interact with the core pluripotency factors NANOG, OCT4, and SOX2, to maintain the stem cell state and to inhibit differentiation. Depletion of Tbx3 leads to differentiation of murine pluripotent stem cells [13]. Moreover, Tbx3 upholds pluripotency by acting as a downstream activator of WNT signaling and plays a role in the reprogramming process by direct binding and activation of the Oct4 promoter. During early differentiation processes, Tbx3 is involved in mesendodermal differentiation, heart development, and limb formation [14]. Additionally, Tbx3 is highly expressed in definite endoderm progenitors and together with Jmjd3 and Eomes promotes the formation of the endoderm [15]. In that respect, Tbx3 was classified as one of the mesendoderm pluripotency transcription factors.

Based on these findings, we applied a knockdown of* TBX3* in human somatic cells to investigate the role of TBX3 in the reprogramming process.

## 2. Materials and Methods

### 2.1. Keratinocyte and Fibroblast Cultivation

The cultivation of keratinocytes from plucked human hair was mainly performed according to [16]. In brief, keratinocytes were cultured on 20 *μ*g/mL collagen IV (Sigma-Aldrich) coated dishes in EpiLife medium with HKGS supplement (both from Gibco) until they reached ~70% confluency.

Human foreskin fibroblasts (HFFs) (System Biosciences) were cultivated in Dulbecco's modified Eagle's medium (DMEM) supplemented with 10% fetal bovine serum (FBS) and 1% Glutamax, 1% nonessential amino acids (NEAA), and 1% antibiotic-antimycotic (all from Life Technologies).

### 2.2. Lentivirus Production, Infection, and Selection

4 × 10^6^ Lenti-X 293T cells (Clontech) were transfected with 8 *μ*g DNA of plasmid pRRL.PPT.SF.hOKSMco.idTom.pre FRT [17], or TRIPZ Human TBX3 shRNA (V2THS_135043, GE Healthcare) together with 2 *μ*g pMD2.G and 5.5 *μ*g of psPAX2 vector DNA (Addgene 12259 and 12260 from Didier Trono) using 1 *μ*g/mL polyethylenimine (PEI). Lenti-X Concentrator Kit (Clontech) was used to concentrate the viral supernatant collected after two and four days. Virus pellets were resuspended in EpiLife with HKGS supplement. Spinfection with 1000 g for 30 min was used to transduce TRIPZ TBX3 lentivirus into the cells on two consecutive days with a medium change after 4 h, respectively. Infected cells were selected by puromycin (1 *μ*g/mL) treatment for two days.

### 2.3. Feeder Cells, Reprogramming of Keratinocytes and Fibroblasts

Rat embryonic fibroblasts (REFs) from embryonic day 14 were generated according to the protocol previously described in [18] and were cultured in DMEM containing 10% fetal bovine serum (FBS), 1% Glutamax, 1% NEAA, and 1% antibiotic-antimycotic (all from Life Technologies). REFs were treated with 7.5 *μ*g/mL mitomycin C (Biomol) for 2.5 hours for mitotic inactivation.

Keratinocytes and fibroblasts were infected as mentioned above over two days with 5 × 10^8^ lentiviral particles in the appropriate medium containing 8 *μ*g/mL polybrene (Sigma-Aldrich). 1 *μ*g/mL doxycycline was added to the keratinocytes and fibroblasts depending on the condition from the first day of infection.

Two days after infection, keratinocytes and fibroblasts were detached using TrypLE Express (Life Technologies) and were seeded on 1.5 × 10^5^ mitotic inactive REF cells in iPSC medium (DMEM F12 supplemented with 20% knockout serum replacement, 1% antibiotic-antimycotic, 1% NEAA, 1% Glutamax, 100 *μ*M *β*-mercaptoethanol, 10 *μ*M Y-27632, 50 *μ*g/mL ascorbic acid, and 10 ng/mL FGF2). Incubation conditions for reprogramming cells were 37°C, 5% CO_2_, and 5% O_2_. Reprogramming cells were cultured on feeder cells until iPSC colonies were clearly visible and were then mechanically picked and transferred onto Matrigel-coated (Corning) cell culture dishes for feeder-free culture.

### 2.4. Human iPSC Culture, Germ Layer Differentiation

iPSCs were cultivated under feeder-free conditions in FTDA medium according to [19]. For further passaging and germ layer differentiation, iPSCs were detached using ReLeSR (Stemcell Technologies) according to the manufacturer's manual. To form embryoid bodies, detached cell clumps were cultured in suspension using ultralow attachment flasks (Corning) for 7 days. After seeding on Matrigel-coated dishes, the embryoid bodies were cultured two additional weeks adherently. iPSC medium was used for germ layer differentiation.

### 2.5. Immunocytochemistry and Alkaline Phosphatase Staining

Cells were fixed for 15 minutes with 4% paraformaldehyde (PFA) and 10% sucrose. Pluripotency staining was performed using StemLight Pluripotency Antibody Kit according to the manufacturer's recommended conditions (Cell Signaling) with Alexa secondary antibodies (all from Abcam). For germ layer differentiation immunocytochemistry stainings, fixed cells were permeabilized with 0.2% Triton-X for 5 minutes, followed by blocking with 5% bovine serum albumin (BSA) for 60 minutes. Antibodies were used as described below: mouse anti-DESMIN (DAKO, 1 : 500), goat anti-SOX17 (R&D Systems, 1 : 500), rabbit anti-TUBB3 (Covance, 1 : 2000), and Alexa secondary antibodies (all from Abcam). Cells were mounted with ProLong Gold Antifade Reagent with 4′,6-diamidino-2-phenylindole (DAPI) (Life Technologies).

Cells for alkaline phosphatase (AP) staining were fixed in 4% PFA for two minutes. Nitroblue tetrazolium/5-bromo-4-chloro-3-indolyl phosphate (NBT/BCIP) solution was prepared according to the manufacturer's protocol (Sigma-Aldrich). The cells were incubated with the substrate solution for 20 minutes and the reaction was stopped with phosphate-buffered saline (PBS). Pictures were taken under artificial light with a normal camera with particular caution on the same acquisition settings and exposure time. Colony numbers were counted manually or quantified with the ImageJ software by measuring the total intensity in the well area after subtraction of the background value.

### 2.6. Quantitative Reverse Transcription Polymerase Chain Reaction

Total RNA was isolated from cell lysates using RNeasy MiniKit (Qiagen). QuantiTect primer assays were used for all experiments (Qiagen, Supplementary Table 1 in Supplementary Material available online at http://dx.doi.org/10.1155/2016/6759343).

PCR was performed on a StepOne instrument (Applied Biosciences) using QuantiFast SYBR Green RT-PCR Kit (Qiagen) according to the manufacturer's protocol. Relative gene expression was calculated as a ratio of target gene concentration to the housekeeping gene hydroxymethylbilane synthase (*HMBS*) concentration.

## 3. Results

### 3.1. Reprogramming of Human TBX3 Knockdown Cells

**Figure 1:**
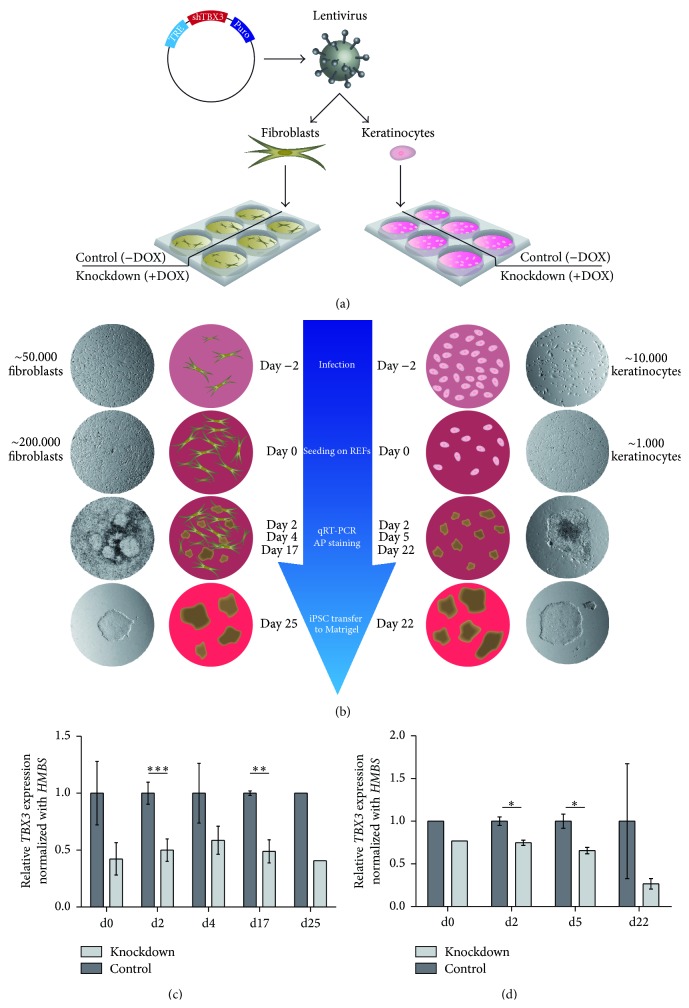
Reprogramming fibroblasts and keratinocytes with a* TBX3* knockdown. (a) Infection of fibroblasts and keratinocytes with a TRIPZ Human* TBX3* shRNA lentivirus, inducible via doxycycline addition. (b) Reprogramming time scheme with fibroblasts (left) and keratinocytes (right) along with starting cell numbers and experiment time points. (c)* TBX3* knockdown during reprogramming of fibroblasts at day 0 (seeding infected cells on feeder layer), day 2, day 4, day 17, and day 25 (last day on feeder cells before transfer to feeder-free system on Matrigel). Control is not treated with doxycycline. (d)* TBX3* knockdown during reprogramming of keratinocytes at day 0 (seeding infected cells on feeder layer), day 2, day 5, and day 22 (last day on feeder cells before transfer to feeder-free system on Matrigel). Control is not treated with doxycycline.

To rule out cell type specific effects in our experimental setup, we chose somatic cells which originate from different germ layers: mesodermal human foreskin fibroblasts (HFFs) and ectodermal human hair follicle keratinocytes. First, we infected both cell types with a lentiviral vector containing a puromycin resistance for selection of infected cells as well as a doxycycline (DOX) inducible shRNA directed against* TBX3* (Figure 1(a)). After successful puromycin selection, cells were either treated with doxycycline (+DOX) or left untreated as a control (−DOX). Subsequently, a defined number of all cell types were infected with a reprogramming lentivirus, containing* OCT4*,* SOX2*,* KLF4,* and C-*MYC*. After two days, infected cells were transferred onto mitotically inactivated feeder cells (rat embryonic fibroblasts). At indicated time points, RNA samples were collected and alkaline phosphatase (AP) stainings were performed (Figure 1(b)).* TBX3* knockdown efficiency was traced throughout the reprogramming process by qRT-PCR. For HFFs, the knockdown was ~50% (Figure 1(c)) while keratinocytes displayed an initial knockdown of approximately 30% showing an increase at later time points (Figure 1(d)). However, it must be taken into account that all measured cell populations contained a considerable amount of rat feeder cells. Due to the highly conserved sequence in both species, the feeder cells partly contribute to the measured* TBX3* levels. Therefore, knockdown efficiency in the reprogramming cells might be considerably higher.

### 3.2. TBX3 Knockdown Reduces Reprogramming Efficiency

**Figure 2:**
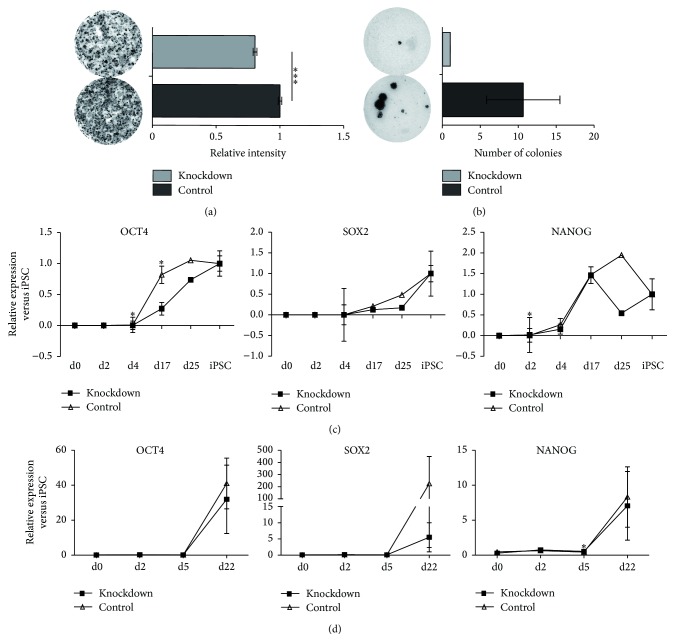
*TBX3* knockdown in fibroblasts and keratinocytes during reprogramming causes decreased reprogramming efficiency. (a) Alkaline phosphatase (AP) staining at day 17 of fibroblast reprogramming with a highly significant reduction (23%) of iPSC colonies. (b) Alkaline phosphatase (AP) staining at day 22 of keratinocyte reprogramming with a reduction (91%) of iPSC colonies. (c) Relative expression of* OCT4*,* SOX2,* and* NANOG* during fibroblast reprogramming. (d) Relative expression of* OCT4, SOX2,* and* NANOG* during keratinocyte reprogramming.

**Figure 3:**
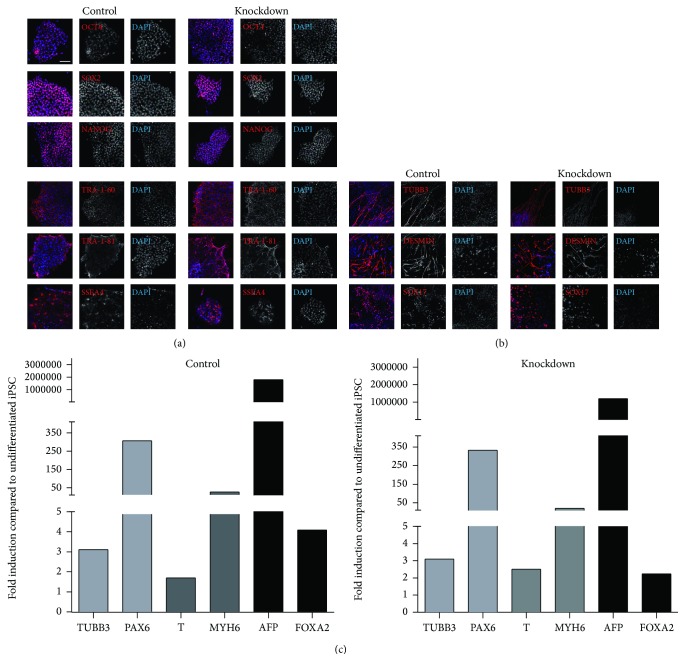
Reprogrammed fibroblasts under* TBX3* knockdown have the same stem cell properties as control iPSCs. (a) Pluripotency staining of iPSCs generated from fibroblasts is positive for the pluripotency markers OCT4, SOX2, and NANOG as well as for the surface markers TRA-1-60, TRA-1-81, and SSEA4. (b) Immunocytochemistry of germ layer differentiation shows positive staining for ectoderm (TUBB3), mesoderm (DESMIN), and endoderm (SOX17) for* TBX3* knockdown and control, respectively. (c) Expression of ectodermal genes (*TUBB3, PAX6*), mesodermal genes (*T, MYH6*), and endodermal genes (*AFP, FOXA2*) in germ layer differentiation shows pluripotent differentiation potential of the generated iPSCs in both control and* TBX3* knockdown cells. Scale bar: 100 *μ*m.

At the end of the reprogramming process, when large iPS colonies were clearly visible, AP stainings were performed and the stained areas were measured. In both cell types, the reduced levels of* TBX3* led to a reduced number of colonies, 23% in HFF (highly significant) and 91% in keratinocytes (Figures 2(a) and 2(b)). Of note, only a very low number of keratinocytes were infected and reprogrammed (due to slow proliferation after infection) leading to a highly reduced number of iPS colonies compared to HFFs which divide continuously and fast during the reprogramming process. Neither colony size nor colony morphology was noticeably altered in knockdown cultures. The reduced reprogramming efficiency was confirmed by qRT-PCR. The RNA levels of* OCT4*,* SOX2,* and* NANOG* were additionally reduced in both TBX3 knockdown cell types, most probably due to the lower number of reprogrammed cells (Figures 2(c) and 2(d)). In later passage iPSCs, RNA levels for* OCT4*,* SOX2,* and* NANOG* levels were comparable to control cultures. Additionally, knockdown cells at later passages displayed a comparable growth speed (not shown) and colony morphology. To generally test the full pluripotent capacity of the knockdown iPSCs, we analyzed later passage HFF derived iPSCs in more detail in terms of pluripotency factor expression and differentiation potential. iPSCs were positive for the transcription factors OCT4, SOX2, and NANOG as well as the surface antigens TRA-1-60, TRA-1-81, and SSEA4 in immunofluorescence staining (Figure 3(a)), comparable to the control cells. In an* in vitro* differentiation experiment, knockdown iPSCs generated cells positive for TUBB3 as an ectodermal marker, DESMIN as a mesodermal marker, and SOX17 as an endodermal marker as shown by immunofluorescence staining (Figure 3(b)). This ability to differentiate into all three germ layers could be underlined by qRT-PCR (Figure 3(c)). Comparable expression levels for different differentiation markers between knockdown and control cells showed that the knockdown does not lead to an altered germ layer preference or diminished differentiation potential. Taken together, the* TBX3* knockdown significantly reduced the reprogramming efficiency but the still arising iPSCs were comparable to control iPSCs.

## 4. Discussion

TBX3 is a core member of the pluripotency network of transcription factors which keeps ESCs and iPSCs in the pluripotent state. Therefore, we were interested if a knockdown of* TBX3* expression would affect the reprogramming process or alter the resulting stem cells in their characteristics. For this, we used two human cell types, fibroblasts and keratinocytes, containing an inducible shRNA directed against* TBX3*. Reprogramming of these cells did not show altered reprogramming kinetics but a reduced reprogramming efficiency compared to control cells. This result was similar for both tested cell types. The number of arising colonies was significantly smaller than in control AP stainings. RNA levels of pluripotency factors were also reduced during the reprogramming process, indicating lower pluripotent cell numbers. Fully reprogrammed cells of later passages, however, did not exhibit altered RNA levels of pluripotency factors. They were also comparable to controls in stem cell marker immunofluorescence stainings. After induction of differentiation, they expressed markers of the three germ layers in a ratio comparable to control cells, without altered germ layer preference. This stays in contrast to a previous study indicating that* TBX3* knockdown inhibits neural rosette formation [20]. However, since we solely investigated RNA levels and not the appearance of neural rosettes, a disturbed rosette morphology cannot be excluded. Interestingly, we found that although the knockdown of* TBX3* was very constant throughout the reprogramming process, in later passage iPSCs it was not visible any more. Here, we suppose that the shRNA does not reduce the very low expression levels any further or that iPSCs may be able to silence the shRNA expression after several passages.

In the future, studies could investigate if the reduction of reprogramming efficiency, as observed with* TBX3* knockdown cells, will also be present in* TBX3* depleted cells or if these cells even fail to be reprogrammed at all. If stable iPSCs can be obtained in this way, their differentiation behavior should be studied in detail since TBX3 is well known to also have important contributions in certain differentiation pathways.

## Supplementary Material

QuantiTect primer assays and the ordering number from Qiagen.
