# Supplementary material for: TBX3 Knockdown Decreases Reprogramming Efficiency of Human Cells
Source: Stem Cells Int. 2015 Nov 30;2016:6759343. doi: 10.1155/2016/6759343 (PMC4677243; doi:10.1155/2016/6759343)
Supplement: Supplementary file 1 — QuantiTect primer assays and the ordering number from Qiagen. [file 6759343.f1.pdf]

Supplementary Table S1:

QuantiTect primer assays:

Gene	Ordering Number
AFP	QT00085183
FOXA2	QT00212786
HMBS	QT00014462
MYH6	QT00030807
NANOG	QT01025850
OCT4	QT00210840
PAX6	QT00071169
SOX2	QT00237601
T	QT00062314
TBX3	QT00022484
TUBB3	QT00083713
